# Highlights from the 2023 International Meeting on the Molecular Biology of Hepatitis B virus

**DOI:** 10.1099/jgv.0.001978

**Published:** 2024-05-16

**Authors:** HBV2023, Kiyasha Padarath, Monika Mani, Laurent Lam, Marion Delphin, Layla Al-Yasiri, Fuwang Chen, Kaho Shionoya, Hu Zhang, Yumeng Li, Jay Basile, Nazim Sarica, Che-Min Lo, Yingchen Zhen, William Schneider, Chi-Ling Hsieh, Hsin-Ni Liu, Sheng-Han Wang, Xiaoming Cheng, Yu Su, Stephanie Maya, Shangqing Yang, Sheng Shen, Sonal Garg, Joseph CY Wang, Nuruddin Unchwaniwala, Ryuta Kojima, Hsin-Ni Liu, Kento Fukano, Tomasz I. Michalak, Chiao-Ling Li, Joao Dias, Maraake Tadesse, Johan Ringlander, James Harris, Gabriela Wu, Cameron Soulette, Loghman Salimzadeh, Nina Le Bert, Hannah Wintersteller, Emily Springer, Valentina Venzin, Xiaolan Xu, Cristian Beccaria, Margaret Chen, Edanur Atez Oz, Anna Kosinska, Francesco Andreata, Marc Windisch, Philip Meuleman, Talisa Richardt, Masaya Funaki, Atsuto Kusonoki, Florian Lempp, Qiong Zhao, Laura McCoullough, Anglero Rodriguez, Helene Strick-Marchand, Adam Zlotnic, Maria Pfefferkorn, Braden Fallon, Pierre Khalfi, Stéphanie Tomba Ngangas, Gnimah Eva Gnouamozi, Johanna bauer, Lena Allweiss, Huanting Chi, Yun-Hua Lin, Simone Fonte, Pei-Yi (Alma) Su, Lena Allweiss, Chari Cohen, Joao Dias, Valeria Fumagalli, Haitao Guo, James M. Harris, Jianming Hu, Matteo Iannacone, Masanori Isogawa, Wen-Juei Jeng, Kyun-Hwan Kim, Anna Kramvis, Wenhui Li, Julie Lucifora, Masamichi Muramatsu, Christine Neuveut, Alexander Ploss, Teresa Pollicino, Ulrike Protzer, Anthony Tan, Yasuhito Tanaka, Thomas Tu, Senko Tsukuda, Robert Thimme, Stephan Urban, Koichi Watashi, Zhenghong Yuan, Shiou-Hwei Yeh, Jane A. McKeating, Peter A. Revill

**Affiliations:** 1 Hepatitis Virus Diversity Research Unit, Department of Internal Medicine, School of Clinical Medicine, Faculty of Health Science,University of Witwatersrand, Johannesburg, South Africa; 2 Department of Medicine, Johns Hopkins University School of Medicine, Baltimore, MD, USA; 3 Sorbonne Universite, UPMC, Paris, France; 4 The Francis Crick Institute, London, UK; 5 Cumming School of Medicine, University of Calgary, Calgary, Canada; 6 Institute of Virology, Technical University of Munich/Helmholtz Zentrum Munchen and German Center for Infection Research (DZIF), partner site Munich, Munich, Germany; 7 National Institute of Infectious Diseases, Toyama, Japan; 8 Cancer Viroloy Program, UPMC Hillman Cancer Center, University of Pittsburgh School of Medicine, Pittsburgh, PA, USA; 9 Key Laboratory of Medical Molecular Virology (MOE/NHC/CAMS), School of Basic Medical Sciences, Shanghai Medical College, Fudan University, Shanghai, PR China; 10 Laboratoire de Virologie Moléculaire, Institut de Génétique Humaine, CNRS, Université de Montpellier, 34000 Montpellier, France; 11 Institut de Génétique Humaine, CNRS, Université de Montpellier, 34000 Montpellier, France; 12 Department of Pathology, John Hopkins University School of Medicine, Baltimore, MD, USA; 13 Wuhan University, Wuhan, PR China; 14 Laboratory of Virology and Infectious Disease, The Rockefeller University, New York, NY, USA; 15 Graduate Institute of Clinical Medicine, National Taiwan University College of Medicine, Taipei, Taiwan, ROC; 16 Baruch S. Blumberg Institute, Doylestown, PA, USA; 17 Hepatitis Research Center National Taiwan University Hospital, Taipei, Taiwan, ROC; 18 Liver Diseases Branch, National Institute of Diabetes and Digestive and Kidney Diseases (NIDDK), National Institutes of Health, Bethesda, MD, USA; 19 Key Laboratory of Medical Molecular Virology (MOE/NHC/CAMS), School of Basic Medical Sciences, Shanghai Medical College, Fudan University, Shanghai, PR China; 20 Princeton University, Princeton, USA; 21 German Cancer Research Center (DKFZ), Virus-Associated Carcinogenesis, Heidelberg, Germany & Heidelberg University, Department of Infectious Diseases, Molecular Virology, Center for Integrative Infectious Disease Research, Heidelberg, Germany; 22 Cancer Viroloy Program, UPMC Hillman Cancer Center, University of Pittsburgh, Pittsburgh, PA 15213, United States & State Key Laboratory of Organ Failure Research, Guangdong Provincial Key Laboratory of Viral Hepatitis Research, Department of Infectious Diseases, Nanfang Hospital, Southern Medical University, Guangzhou, 510515, PR China; 23 Department of Microbiology and Immunology The Pennsylvania State College of Medicine, Hershey, Pennsylvania 17033, USA; 24 The Pennsylvania State College of Medicine, Hershey, Pennsylvania 17033, USA; 25 Assembly Biosciences Inc.,South San Francisco, California, USA; 26 Department of Gastroenterology, Graduate School of Medicine, Chiba University, Chiba 260-8670, Japan; 27 Department of Virology II,, National Institute of Infectious Diseases, Tokyo, Japan; 28 Molecular Virology and Hepatology Research Group, Division of BioMedical Sciences, Faculty of Medicine, Health Sciences Centre, Memorial University of Newfoundland, St. John's, NL, Canada; 29 National Taiwan University, Taipei, Taiwan, ROC; 30 Laboratoire de Virologie Moléculaire, CNRS Université de Montpellier, Montpellier, France; 31 Johns Hopkins University, Department of Medicine, Johns Hopkins University School of Medicine, Baltimore, Maryland, USA; 32 Department of Infectious Diseases, Institute of Biomedicine, Sahlgrenska Academy, University of Gothenburg, Göteborg, Sweden; 33 Nuffield Department of Medicine, Oxford, UK; 34 Storr Liver Centre, The Westmead Institute for Medical Research, The University of Sydney at Westmead Hospital, Westmead, NSW, Australia; 35 Gilead Sciences, Inc., Foster City, California, USA; 36 Toronto Centre for Liver Disease, Toronto General Hospital Research Institute (TGHRI), University Health Network, Toronto, Canada; 37 DUKE-NUS Medical School, Singapore, Singapore; 38 Institute of Molecular Immunology, Technical University of Munich, Munich, Germany; 39 San Raffaele Research Institute, Milano, Italy; 40 Key Laboratory of Medical Molecular Virology, Shanghai Institute of Infectious Diseases and Biosafety, School of Basic Medical Sciences, Shanghai Medical College, Fudan University, Shanghai, PR China; 41 Department of Dental Medicine and Department of Laboratory Medicine, Karolinska Institutet, Solna, Sweden; 42 Institute of Virology, Technical University /Hemholtz Center Munich, Oberschleißheim, Germany; 43 Assembly Biosciences Inc, California, USA; 44 University of Ghent, Ghent, Belgium; 45 University of Heidelberg, Heidelberg, Germany; 46 Kanazawa University Hospital, Kanazawa, Japan; 47 National Institute of Infectious Diseases, Toyama, Japan; 48 Vir Bio, San Francisco, CA, USA; 49 Blumberg Institute, Pennsylvania, USA; 50 Victorian Infectious Diseases Reference Laboratory, Royal Melbourne Hospital, at the Peter Doherty Institute for Infection and Immunity & Department of Microbiology and Immunology, Melbourne, Australia; 51 Chroma Medicine / TIGET, Boston, MA, USA; 52 Institute Pasteur, Paris, France; 53 Indiana University, Bloomington, Indiana; 54 Division of Hepatology, Department of Medicine II, Leipzig University Medical Center, Leipzig, Germany; 55 University of Utah, Salt Lake City, UT, USA; 56 Institut de Genetique Moleculaire de Montpellier, University of Montpellier, CNRS, France; 57 Laboratoire de microbiologie medicale, hopital Avicenne (AP-HP), Centre national de reference des virus des hepatites B, C et Delta, hopital Avicenne (AP-HP), Unit. INSERM U955, equipe no18, Universite Paris Est, Créteil, France; 58 Department of Infectious Diseases, Molecular Virology, Heidelberg University, Heidelberg, Germany; 59 University of Heidelberg Centre for Infectious Diseases, Heidelberg University Hospital, Heidelberg, Germany; 60 University Medical Center Hamburg-Eppendorf, German Center for Infection Research (DZIF), Hamburg, Germany; 61 Department of Infectious Diseases, Virology, University Hospital Heidelberg and German Centre for Infection Research (DZIF), Partner Site Heidelberg, Heidelberg, Germany; 62 Department and Graduate Institute of Microbiology, College of Medicine, National Taiwan University, Taipei, Taiwan, ROC; 63 Cancer Research Center of Lyon (CRCL), INSERM U1052, CNRS UMR5286, Institute d’Hepatologie de Lyon, Lyon, France; 64 Institute of Biomedical Sciences, Academia Sinica, Taipei, Taiwan, ROC; 1 Various- see supplementary material; 2 Department of Internal Medicine, University Medical Center Hamburg-Eppendorf, Hamburg, Germany; 3 German Center for Infection Research (DZIF), Braunschweig, Germany; 4 Hepatitis B Foundation, Doylestown, PA, USA; 5 Laboratoire de Virologie Moléculaire, CNRS Université de Montpellier, Montpellier, France; 6 Division of Immunology, Transplantation and Infectious Diseases, IRCCS San Raffaele Scientific Institute, 20132 Milan, Italy; 7 Vita-Salute San Raffaele University, 20132 Milan, Italy; 8 Department of Microbiology and Molecular Genetics; Cancer Virology Program, UPMC Hillman Cancer Center, University of Pittsburgh School of Medicine, Pittsburgh, Pennsylvania, USA; 9 Nuffield Department of Medicine, University of Oxford, Oxford, UK; 10 Department of Microbiology and Immunology, The Pennsylvania State University College of Medicine, Hershey, PA, USA; 11 Department of Virology II, National Institute of Infectious Diseases, Tokyo, Japan; 12 Department of Gastroenterology and Hepatology, Chang Gung Memorial Hospital, Linkou Medical Center, Chang Gung University, Taoyuan, Taiwan, ROC; 13 Department of Precision Medicine, School of Medicine, Sungkyunkwan University, Suwon, Republic of Korea; 14 Anna Kramvis Hepatitis Virus Diversity Research Unit, Department of Internal Medicine, School of Clinical Medicine, University of the Witwatersrand, Johannesburg, South Africa; 15 National Institute of Biological Sciences, Beijing, PR China; 16 Tsinghua Institute of Multidisciplinary Biomedical Research, Tsinghua University, Beijing, PR China; 17 CIRI, Centre International de Recherche en Infectiologie, Univ Lyon, Inserm, U1111, Université Claude Bernard Lyon 1, CNRS, UMR5308, ENS de Lyon, F-69007, Lyon, France; 18 Department of Infectious Disease Research, Institute of Biomedical Research and Innovation, Foundation for Biomedical Research and Innovation at Kobe, Kobe, Hyogo, Japan; 19 Department of Molecular Biology, Princeton University, Princeton, NJ, USA; 20 Department of Clinical and Experimental Medicine, University Hospital of Messina, Messina, Italy; 21 Institute of Virology, School of Medicine and Health, Technical University of Munich/Helmholtz Munich, Munich, Germany; 22 Emerging Infectious Diseases, Duke-NUS Medical School, Singapore, Singapore; 23 Department of Gastroenterology and Hepatology, Faculty of Life Sciences, Kumamoto University, Kumamoto 860-8556, Japan; 24 Storr Liver Centre, The Westmead Institute for Medical Research, The University of Sydney at Westmead Hospital, Westmead, NSW, Australia; 25 Centre for Infectious Diseases and Microbiology, Sydney Infectious Diseases Institute, The University of Sydney at Westmead Hospital, Westmead, NSW, Australia; 26 Department of Medicine II, University Hospital Freiburg, Breisgau, Germany; 27 Research Center for Drug and Vaccine Development, National Institute of Infectious Diseases, Tokyo, Japan; 28 Key Lab of Medical Molecular Virology, Shanghai Medical College, Fudan University, Shanghai, PR China; 29 Graduate Institute of Microbiology, National Taiwan University College of Medicine, Taipei, Taiwan, ROC; 30 Department of Clinical Laboratory Sciences and Medical Biotechnology, National Taiwan University College of Medicine, Taipei, Taiwan, ROC; 31 Chinese Academy of Medical Sciences Oxford Institute, University of Oxford, Oxford, UK; 32 Victorian Infectious Diseases Reference Laboratory, Royal Melbourne Hospital at the Peter Doherty Institute for Infection and Immunity, Parkville, Victoria, Australia; 33 Department of Infectious Diseases, University of Melbourne, Parkville, Victoria, Australia

**Keywords:** International HBV Meeting 2023

## Abstract

Since its discovery in 1965, our understanding of the hepatitis B virus (HBV) replication cycle and host immune responses has increased markedly. In contrast, our knowledge of the molecular biology of hepatitis delta virus (HDV), which is associated with more severe liver disease, is less well understood. Despite the progress made, critical gaps remain in our knowledge of HBV and HDV replication and the mechanisms underlying viral persistence and evasion of host immunity. The International HBV Meeting is the leading annual scientific meeting for presenting the latest advances in HBV and HDV molecular virology, immunology, and epidemiology. In 2023, the annual scientific meeting was held in Kobe, Japan and this review summarises some of the advances presented at the Meeting and lists gaps in our knowledge that may facilitate the development of new therapies.

## Introduction

**Fig. 1.**
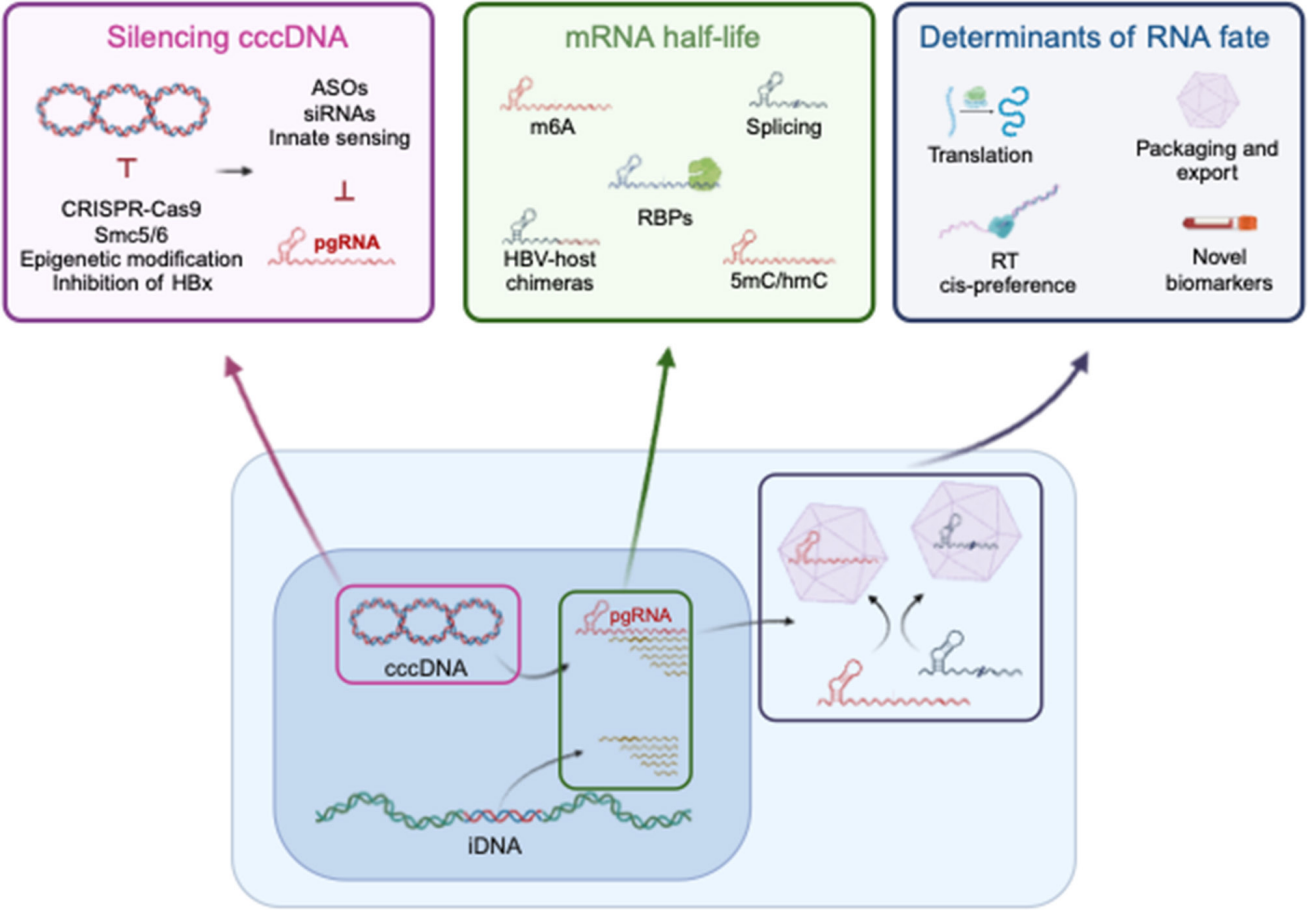
HBV replication life cycle and open questions. A cartoon depicting topics summarised in this article, highlighting some of the currently unknown features of the HBV life cycle. cccDNA, covalently closed circular DNA; iDNA, integrated HBV DNA; pgRNA, pre-genomic RNA; Smc5/6, structural maintenance of chromosomes 5/6 complex; HBx, hepatitis B virus X protein; ASOs, anti-sense oligonucleotides; m6A, N6-methyladenosine; RBPS, RNA-binding proteins; 5mC/hmC 5-methylcytosine/hydroxymethylcytosine.

Hepatitis B virus (HBV) is the prototype member of the family *Hepadnaviridae*, encoding a 3.2 kb genome of relaxed circular partially double-stranded DNA (rcDNA). The HBV genome was first cloned in 1979 and decades of research have revealed a complex replication cycle where capsids traffic to the nucleus and genomes are repaired to generate a covalently closed circular DNA (cccDNA) transcriptional template for the genesis of viral RNAs [1,2]. Nonetheless, gaps in knowledge concerning key aspects of the HBV replication cycle remain, particularly regarding the biogenesis of cccDNA and host pathways regulating its transcriptional activity (Fig. 1). Additional areas of investigation include the kinetics and location of HBV nucleocapsid uncoating; factors regulating pregenomic RNA (pgRNA) splicing; encapsidation versus translation; assembly and trafficking of complete virion (Dane) particles versus subviral particles (SVPs); and the role of hepatitis B surface antigen (HBsAg), HBx and HBe antigens in the replicative life cycle and immune response (Table 1).

**Table 1.** Knowledge gaps and challenges for the HBV field

Knowledge gaps
**HBV replication**
Capsid uncoating – timing and location
Biogenesis and stability of cccDNA
Pathways regulating cccDNA transcription
pgRNA splicing, encapsidation vs translation
Assembly and trafficking of Dane particles versus subviral particles
Contribution of cccDNA and integrated viral HBV to protein expression
Basis for HBV’s narrow species tropism
**HBV immunology**
Role of L, M and S components of the HBsAg
Factors controlling immune dysfunction
Factors controlling HBeAg and HBsAg seroconversion
Dynamics of CD4 T cell and B cell activation
HBV evasion/subversion of innate immune responses
**Cell biology**
Impact of HBV infection on host transcriptome, proteome and metabolome
Mechanisms by which HBV causes HCC
Role of genotypes and subgenotypes of HBV on disease progression
**Antivirals**
How to stimulate host antiviral immune responses
Understanding maintenance of HBsAg loss

Chronic hepatitis B (CHB) is a global public health threat, with an estimated 296 million people affected [3,4]. Chronic infection is associated with significant morbidity and mortality resulting from end-stage liver disease, including cirrhosis and hepatocellular carcinoma (HCC). HCC has the seventh highest age-adjusted cancer incidence rate globally and is the third highest cause of cancer-related mortality [5,6]. However, the mechanisms by which HBV causes liver cancer are not well understood. HBV is not cytopathic and liver damage is largely due to immune-mediated clearance of HBV-infected cells; however, our understanding of T and B cell-mediated immune responses to HBV, including factors driving immune exhaustion, are not well defined (Table 1). How HBV evades or subverts host innate immune responses is an active area of research. Our understanding of the impact of HBV infection on the host transcriptome, proteome and metabolome is lacking. Currently there are no curative treatments for individuals living with HBV; the preventative vaccine has no effect on existing infections and current therapies only suppress viral replication and have a limited impact on viral RNA and antigen expression. Interferon alpha (IFNα) is the oldest approved therapy and can have severe side effects, limiting compliance, and is effective in a minority of patients. The HBV-encoded polymerase has reverse transcriptase activity and is the target for current nucleoside therapies. These drugs block the *de novo* synthesis of HBV DNA but do not target the cccDNA reservoir, meaning there is a lifelong risk of viral rebound if therapy is stopped.

Spontaneous or clinical resolution of chronic infection and/or acute hepatitis is unlikely to eradicate the infection as cccDNA persists [7,9] and may reactivate during immune suppression [10]. HBV persistence is thought to be facilitated by the long-lived reservoir of cccDNA. HBsAg levels in the periphery associate with progressive liver disease and are a bio-marker for assessing new therapies to achieve a functional cure [11], currently defined as HBsAg loss [12]. A full understanding of the HBV replication cycle and associated immune responses will facilitate progress towards this important goal.

**Fig. 2.**
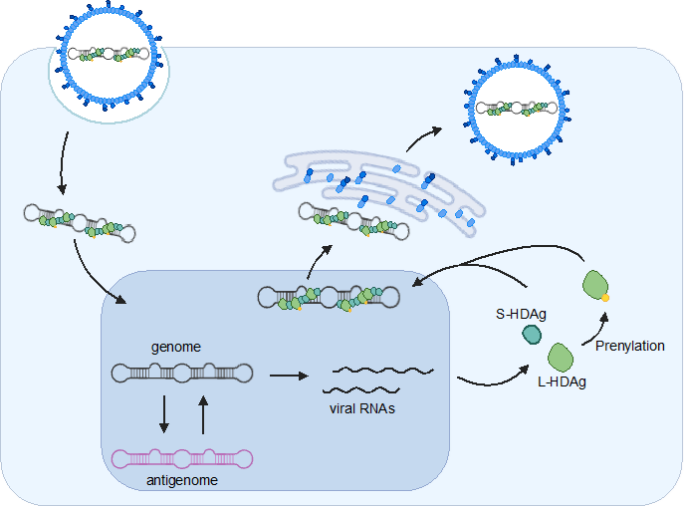
HDV life cycle. S-/L-HDAg, small/large hepatitis D virus antigen.

HDV is a small, enveloped virus with a circular single-stranded RNA genome that does not encode envelope proteins and is dependent on HBV as a helper virus, which provides its surface glycoproteins to produce infectious particles [13,14]. The molecular biology of HDV is unique among animal viruses and RNA genome replicates through a dual-rolling-circle mechanism that employs one viral encoded protein and two ribozymes (Fig. 2). An estimated 15 million people worldwide are chronically infected with HDV and HBV and this co-infection is associated with a higher risk of severe hepatitis, cirrhosis, and HCC [15]. Gaps remain in our knowledge of cellular factors required for HDV replication and HDV regulation of host factors, as well as the interactions between HDV and HBV in the setting of coinfection (reviewed in [16]).

The first conference on the Molecular Biology of Hepatitis B Viruses was held in 1985 at Cold Spring Harbor and was co-organised by Harold Varmus and Jesse Summers; scientists have met annually since then to discuss the latest advances in HBV and HDV research. This review highlights presentations selected by session co-chairs from the 2023 Meeting that address many of the knowledge gaps outlined above.

## HBV diversity and epidemiology

HBV replicates via an error prone reverse transcriptase (RT) that results in sequence heterogeneity that is classified into 9 genotypes, A to I, and 35 sub-genotypes [17]. However, HBV is highly conserved, largely due to the overlapping nature of the genome and the necessity of maintaining the integrity of all three reading frames and regulatory regions. Deep sequencing has revealed hotspots of sequence conservation that may represent novel antiviral targets across HBV genotypes [17,18]. In turn, clinically relevant mutations have been observed, particularly in the RT region, conferring resistance to some direct acting antiviral agents [19,20]. Padarath *et al*. (Kramvis lab, University of Witwatersand) studied the proteome of Huh-7 hepatoma cells transfected with plasmids encoding HBV genotypes A1, A2, D3 and E, and showed reduced expression of proteins involved in inflammation, T cell activation, and apoptosis in genotypes A1, D3, and E compared to A2, suggesting that these differences may contribute to the increased hepatocarcinogenic potential of some viral genotypes. Mani *et al*. (Balagopal lab, Johns Hopkins University) sequenced HBV RNA from approximately >200 cell equivalents using *in situ* transcriptomics and demonstrated multiple viral genotypes with variable frequencies of ‘drug-resistant’ mutations in the polymerase gene. This study highlights potential intracellular diversity generated from a cccDNA pool that is worthy of further investigation.

The real-world clinical and economic burden of CHB was assessed using the French national health insurance database (SNDS). Lam *et al*. (Carrat lab, Sorbonne Université) studied a cohort of HBV-infected patients that was matched with the general population based on birth year and sex and showed an annual excess mortality rate of 13.6 per 1000 individuals, nearly three times the death rate in the general population (14 459 deaths versus 5035). The healthcare expenditures for CHB patients, including both direct and indirect medical costs such as in-patient care, out-patient care, medication, medical devices, laboratory tests, and work-related accidents, amounted to €4.2 billion. This study underscores the importance of implementing efficient screening, prevention, and treatment programmes to reduce the clinical and economic burden of CHB. Delphin *et al*. (Matthews lab, The Francis Crick Institute) highlighted the lack of HBV clinical trials in Africa, where a large CHB population is overlooked [21].

## Early steps in the HBV life cycle – virus entry

**Fig. 3.**
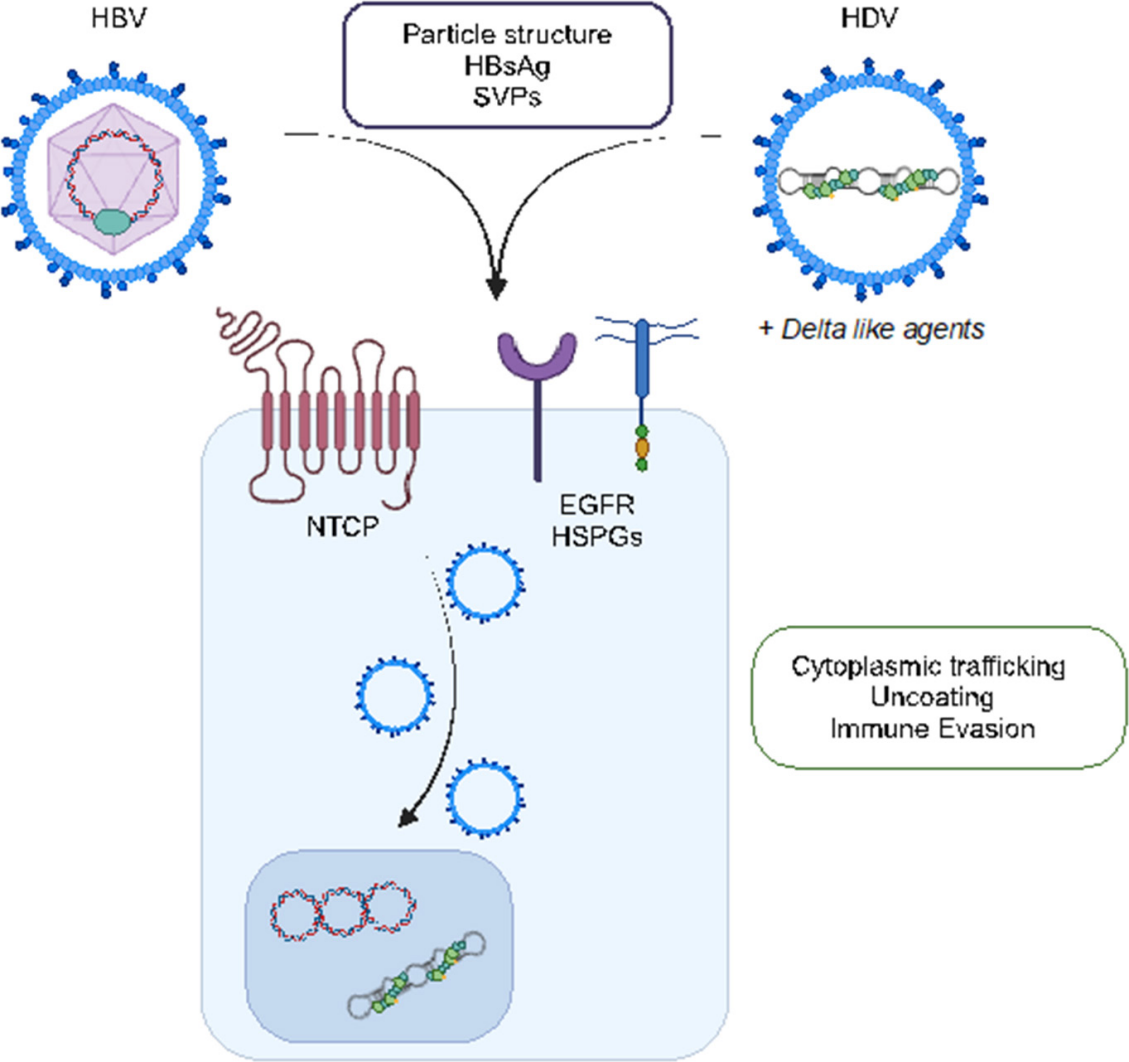
Essential role for NTCP in regulating HBV and HDV entry into hepatocytes. HBV and HDV, as well as delta-like agents, share NTCP as a receptor. The boxes highlight some of the emerging areas of research discussed in this article. NTCP, sodium taurocholate co-transporting polypeptide; EGFR, epidermal growth factor receptor; HSPGs, heparan sulphate proteoglycans; SVPs, subviral particles.

The sodium taurocholate co-transporting polypeptide (NTCP) is recognised as a primary receptor in defining HBV and HDV infection of hepatocytes in the liver (Fig. 3). Our knowledge of the early steps following HBV entry into the liver is limited and Al-Yasiri *et al*. (Coffin lab, University of Calgary) used intravital microscopy to study woodchuck hepatitis virus (WHV) infection. The authors inoculated animals with fluorescent WHV and observed liver-resident macrophages (Kupffer cells) capture virus within minutes of infection, providing a rare insight into the complexities of the liver microenvironment. Chen *et al*. (Protzer lab, Technical University of Munich) studied diverse species of the surface receptor NTCP and showed that the horse analogue of NTCP (hoNTCP) supported a higher susceptibility to HBV infection than human NTCP-expressing hepatoma cells. The authors found that HepG2 hepatoma cells expressing hoNTCP supported high levels of cccDNA formation and this observation may elucidate post-entry steps for NTCP in regulating particle trafficking.

Shionoya *et al*. (presented by Kobayashi, Watashi lab, National Institute of Infectious Diseases, Japan) presented the complex structure of NTCP with preS1 domain of the virally encoded large envelope protein. The authors generated an antibody against recombinant NTCP complexed with myristoylated preS1 peptide consisting of 2–48 aa (myr-preS1) and included the Fab fragment in the NTCP/myr-preS1 complex. Cryo-EM single-particle analysis revealed the NTCP/myr-preS1/Fab structure at 2.89–3.11 Å resolution that showed NTCP adopting a similar conformation to the apo-NTCP structure. In the new structure, the N-terminal region (aa 2–33) of myr-preS1 interacts with the bile acid channel region of NTCP, and the channel is extended upon myr-preS1 binding, while the C-terminal region (aa 36–48) interacts with the extracellular surface of NTCP in the complex. It remains to be determined how the myristylation moiety of the myr-preS1 peptide interacts with NTCP. This study aims to accelerate the development of new inhibitors targeting NTCP.

## HBV cccDNA expression

As cccDNA is essential for maintaining the HBV life cycle, it is an attractive drug target, and reporter model systems that support high-throughput screening (HTS) are in demand. Zhang *et al*. (Guo lab, University of Pittsburgh) presented a novel cccDNA luciferase reporter cell line, HepBLE12, that encodes a split nanoluciferase HiBiT tag in the precore sequence of the HBV transgene. This cell line provides a direct intracellular cccDNA readout and enables HTS of antiviral agents targeting cccDNA. Li *et al*. (Yuan lab, Fudan University) developed an M13 phage-based ssDNA production system coupled with deoxyribozyme that facilitated the rapid genesis of full-length HBV cccDNA without foreign sequences. When transfected in hepatoma cells, the *in vitro*-generated cccDNA transcribed viral RNAs and responded to antiviral drugs, providing a new system for HTS screens of antiviral agents and studying cccDNA biology. Jay *et al*. (Dias Lab, CNRS, France) used mass spectrometry to analyse cccDNA-associated proteins in HBV-infected human hepatocytes and found that H2A.Z.1 (a sub-variant of histone H2A) and two chaperones (SRCAP and p400) associated with cccDNA. H2A.Z has three isoforms in mammals, H2A.Z.1, H2A.Z.2.1 and H2A.Z.2.2, and has been implicated in transcriptional control, DNA repair, regulation of heterochromatin, and cancers. The authors suggest that H2AZ1 may impact on cccDNA establishment and, together with SRCAP, may regulate HBV transcription. Sarica *et al*. (Neuveut lab, Institute of Human Genetics, CNRS, France) developed a multiplex digital droplet PCR assay that allowed simultaneous quantification of HBV transcripts in cell culture models (HepAD38, HepG2-NTCP cells). Primers targeting different regions of the partially overlapping transcripts were validated with synthetic DNA templates with one-step and two-step PCR approaches.

To define the host factors that regulate the abundance of HBV RNAs in the chronically infected liver, Lo *et al*. (Balagopal lab, Johns Hopkins University) characterised gene expression in liver biopsies from HIV co-infected HBeAg^+^ patients. Hepatocytes were grouped as expressing high or low HBV RNAs and differentially expressed genes known to regulate metabolic processes and innate immune responses associated with high levels of HBV transcripts. Identifying how these pathways regulate steady state RNA levels and whether they associate with clinical progressive disease will be of interest. To investigate whether host RNA-binding proteins regulate the nuclear export of HBV transcripts, Zhen *et al*. (Xia lab, Wuhan University) screened the interaction of HBV RNA with host proteins using a quantitative proteomics approach. The group identified embryonic lethal, abnormal vision, Drosophila-like 1 (ELAVL1) as a viral RNA-binding partner [22]. ELAVL1 silencing inhibited the nuclear export of HBV RNA and suppressed viral replication in both cell and mouse models of HBV replication. Mechanistic studies revealed that HBV RNA associates with ELAVL1 to recruit CRM1 after binding to ANP32A and ANP32B and this may regulate pgRNA half-life. The authors conclude that there is a role for ELAVL1 in the regulation of HBV RNA stability and nucleocytoplasmic transport via the CRM1 nuclear export pathway.

Schneider *et al*. (Rice lab, Rockefeller University) used mutational scanning to create a fitness map of the HBV polymerase at single-nucleotide resolution. Conserved proline residues in the unstructured C-terminus were identified and shown to stall ribosomes to favour *cis*-preferential pgRNA packaging. Hsieh *et al*. (Yeh lab, National Taiwan University) reported that dephosphorylation of the viral encoded core protein (HBc) at position S170 was polymerase-dependent and an essential step preceding pgRNA encapsidation. Using mass spectrometry, the authors identified that protein phosphatase 1 was recruited by the viral polymerase to dephosphorylate HBc. Together, these findings advance our knowledge of HBV cccDNA structure and expression, although further work is required to understand factors promoting the biogenesis and maintenance of the cccDNA reservoir critical for HBV persistence.

## HBV–host interplay

The HBV x protein (HBx) regulates cccDNA transcription via recruiting the DDB1-CUL4 complex to degrade SMC5/6 in hepatocytes. Liu *et al*. (Guo lab, University of Pittsburgh) showed that human hepatoma cells commonly used to study HBV replication express low levels of DDB1 and this impacts on the DDB1-CUL4-SMC5/6 nexus. Expressing DDB1 in hepatoma cells restores HBx-mediated SMC5/6 degradation and enhances cccDNA transcriptional activity. The authors noted that the oncogenic poly(ADP-ribose) glycohydrolase (PARG) influences DDB1 stability, and suggested a role for the PARG–DDB1 interplay in HBV infection and liver cancer development. Wang (Wang lab, National Taiwan University) reported that HBx promotes androgen receptor (AR) retention in mouse hepatocytes by inhibiting starvation-induced chaperone-mediated autophagy (CMA) activity. In addition to AR, which has a physiological function in regulating gluconeogenesis, several metabolic enzymes have been identified as CMA substrates. Thus, perturbation of hepatic CMA function may provide a mechanism for HBx to perturb hepatocellular metabolism.

Cheng *et al*. (Liang lab, National Institutes of Health) performed a genome-wide siRNA screen to identify host factors that regulate HBV transcript abundance and discovered a role for neuroblastoma RAS viral (v-ras) oncogene homologue (NRAS). Knocking down NRAS elevated HBV transcripts in HepG2-NTCP cells without affecting cccDNA levels. NRAS depletion enriched lipid metabolism genes and increased intracellular triglycerides and cholesterol. In addition, the authors noted increased expression of HNF4A, a transcription factor previously reported to activate HBV transcription. The authors suggested a role for NRAS to regulate HBV replication through the lipid metabolism–HNF4A axis. Su and Tsai (Tsai lab, Academia Sinica) presented work on the cytidine methylation, m5C, in the epsilon element of pgRNA. The authors showed a role for the cellular RNA methyltransferase NSUN2, and CRISPR-mediated depletion of NSUN2 from infected Huh-7 cells reduced the secretion of HBV particles. Interestingly, this phenotype resulted from reduced HBc protein expression and a loss of reverse transcriptase products, suggesting a role for host factors in regulating HBV reverse transcription. Su *et al*. (Deng lab, Fudan University) developed a transgenic mouse model carrying an HBV genome with basal core promoter mutations. These mice showed increased HBc expression that was associated with disrupted mitochondrial dynamics and lysosomal disturbance and increased cell death, suggesting that HBc can drive liver injury in immunodeficient mice and highlighting HBc as a potential therapeutic target.

Species restriction of HBV remains a barrier towards the development of small and cost-effective animal models. Murine NTCP does not support HBV entry, whereas transgenic mice expressing human NTCP (hNTCP) do not support infection, demonstrating that additional human cellular factors are required for post-entry steps of the virus life cycle. HBV infection is aborted in hNTCP-expressing mouse cells before conversion of the incoming HBV genome into cccDNA. Maya *et al*. (Ploss lab, Princeton University) performed systematic loss-of-function assays of nuclear import-related proteins and identified several candidates that are critical for HBeAg secretion in human cells. Interestingly, overexpression of those candidates in hNTCP-expressing mouse cells led to production of HBV antigens and these insights provide potential transgenes to generate much needed permissive mouse models.

## HBV assembly and high-resolution imaging of particles

Virus assembly is an understudied area of HBV biology and Yang *et al*. (Bartenschlager lab, Heidelberg University) identified host factors associated with HBV and subviral particles. Employing chromatography, ultracentrifugation and immunocapture, the authors purified these particles and identified ~160 host factors. Phenotypic validation identified 18 factors that regulated HBV and subviral particle production. Newly assembled HBV particles are secreted via the endosomal sorting complexes required for the transport (ESCRT)/multivesicular body (MVB) pathway. However, the pathways that mediate the release of naked capsids that lack an envelope are not well understood. Sheng *et al*. (Guo lab, University of Pittsburgh) discovered that a K96R mutation in HBc prevented naked capsid egress whilst having no effect on virion secretion. They demonstrated that HBc ubiquitination at the K96 position by the NEDD4 family ubiquitin ligase AIP4 was required for the release of naked capsids. Garg *et al*. (Wang and Hu lab collaboration, Pennsylvania State University College of Medicine) used cryo-electron microscopy to visualise a site on the exterior of naked capsids where a 22 amino acid region of the pre-S domain of the large surface protein, previously identified as the matrix domain (MD), interacts with capsids during virion formation. Wang *et al*. (in collaboration with Hu lab, The Pennsylvania State University College of Medicine) studied empty hepatitis B virions purified from patient serum by single-particle cryo-EM, providing the first visualisation of the surface protein–capsid interaction, and showed that the lipid components of the virion envelope layer were not organised in a traditional bilayer. Noting the critical role for nucleocapsids in the viral replication cycle, ongoing efforts to resolve high-resolution structures of hepatitis B capsids and virions could help develop new antiviral strategies [23].

## HBV integrants and viral pathogenesis

Global research efforts have focused on mapping integrated HBV DNA, identifying their transcriptional activity and potential role in pathogenesis. Unchwaniwala *et al*. (Assembly Biosciences, Inc.) showed that the new capsid inhibitor ABI-4334 could reduce integration events in a HepG2-NTCP model. This provides an advantage over current RT inhibitors and first-generation capsid inhibitors, which have a minimal impact on integration events in similar experimental infection models [24]. Several investigators reported on new methods to detect HBV integrants. Kojima *et al*. (Kato lab, Chiba University) developed an algorithm (GRIDSS VIRUSBreakend) to map HBV DNA integrations from next-generation sequencing data [25]. Other groups targeted biological sources; Liu *et al*. (Su lab, Baruch S. Blumberg Institute) detected HBV integrants by analysing cell-free DNA in the urine, while Fukano *et al*. (Muramatsu lab, National Center for Global Health and Medicine) studied cell-free DNA in serum. These studies offer new approaches to detect HBV integrants and to study associated disease pathways.

Michalak *et al*. (Michalak lab, Memorial University) observed that WHV integrations occurred within minutes to hours post-infection. Infection of woodchucks was accompanied by an increase of reactive oxygen species and genes associated with DNA repair. Moreover, integration sites early in infection were distinct from those acquired later in infection. Li *et al*. (Yeh lab, National Taiwan University) characterised HBV integrated events in liver biopsies and found that HBV genotype C integrants were enriched at the TERT hotspot and rearrangements were commonly observed in viral sequences. Liu *et al*. (Su lab, Baruch S. Blumberg Institute) used HBV-targeted next-generation sequencing to characterise double-stranded linear DNA (dslDNA)-derived cccDNA in treatment-naïve CHB patients. In all patients, dsl-cccDNA was detected independently of intrahepatic cccDNA levels, with higher frequencies in HBeAg-positive than HBeAg-negative patients. Dias *et al*. (Neuveut lab, CNRS) identified long-range contacts between DNA sequences containing integrated HBV DNA and distal genomic regions enriched for cancer related genes, suggesting that such long-range contacts may contribute to altered cellular phenotypes.

Taddesse *et al*. (Thio lab, Johns Hopkins University) studied the contribution of HBV integrants to HBsAg expression in patients with HBV/HIV coinfection, suggesting that HBsAg expression shifts from cccDNA to integrated HBV DNA during long-term antiviral treatment. Probe enrichment techniques to sequence low-copy-number HBV RNAs in liver tissue samples were reported by Ringlander *et al*. (Lindh lab, University of Gothenburg) and by Harris *et al*. (McKeating lab, University of Oxford), highlighting this approach to accurately map the HBV transcriptome from cccDNA and integrants. Using a long-read sequencing approach, Harris *et al*. identified variable patterns of the preS1, preS2, and S transcripts that encode large (LHBs), middle (MHBs) and small surface protein (SHBs), respectively. Recent clinical reports showing the importance of HBsAg composition in predicting early treatment responses [26,27] underscore the importance of monitoring expression of L/M/S HBs glycoproteins and to understand the pathways regulating their expression.

Wu *et al*. (Tu lab, University of Sydney) presented data that HBe and HBc could be expressed from integrated HBV DNA in *in vitro* infection systems, where cells carrying HBV integrants that were devoid of cccDNA expressed HBe and HBc antigens. This was consistent with long-read genome and transcriptome sequencing data from liver biopsies from HBV-infected patients, presented by Soulette *et al*. (Gilead Biosciences). Together, these studies could challenge the concept of a sterilizing HBV cure to limit viral reactivation.

## Adaptive immunity

There are considerable gaps in our knowledge regarding immune-mediated pathogenesis and control of HBV infection [28]. Several studies explored HBV-specific T cell dysfunction, the modulation of potential co-signalling receptors and how peripheral HBsAg influences T cell populations. Studies from Salimzadeh (Gehring lab, University of Toronto) and Le Bert (Bertoletti lab, Duke-NUS Medical School) delved into the functional variability of HBV-specific T cells across different CHB infection stages. Their data underscored the significance of T cell responses in understanding host–viral dynamics and predicting therapeutic outcomes. Wintersteller *et al*. (Knolle lab, Technical University of Munich) proposed that CXCR6^+^ CD8^+^ T cells, which show similar features and functions in the bloodstream and liver, could be used to monitor liver responses in CHB. Springer *et al*. (Wohlleber lab, Technical University of Munich) presented their work to boost dysfunctional HBV-specific CD8 T cells using type I interferon in a HBV transgenic mouse model. Venzin *et al*. (Iannacone lab, San Raffaele Scientific Institute) presented findings on how HBV-specific CD4^+^ T cells in HBV transgenic mice influence Kupffer cells, which in turn reduce CD8^+^ T cell dysfunction. Presentations by Xu *et al*. (Li lab, Fudan University) and Beccaria *et al*. (Iannoacone lab, San Raffaele Scientific Institute) offered insights into B cell dynamics in persistent HBV exposure and the interactions between HBsAg-specific B cells and CD4^+^ T cells.

Several presentations highlighted the crucial role of HBsAg clearance, representing both the resolution of acute HBV infection and the therapeutic target for functional cure. Specific studies showcased the potential role of PD-1 in early HBV-specific CD8^+^ T cell expansion and a CD3-expressing macrophage population’s contribution to HBV clearance. In terms of immune-based therapies several presentations provided updates on chimaeric antigen receptor designs, MHC-E-restricted CD8^+^ T cells, the characterisation of anti-HBsAg-neutralizing antibodies, and anti-PD-L1-blocking antibodies. Particularly noteworthy was the study by Tajpara *et al*. (Chen lab, Karolinska Institute) on HBV-TCR redirected mucosal-associated invariant T cells (MAIT) cells for HBV-related HCC.

One promising therapy for CHB is therapeutic vaccination. An earlier study showed that vaccination based on a heterologous protein–prime/recombinant MVA-boost scheme, called TherVacB, was efficacious in preclinical mouse models with low-to-intermediate but not high HBV replication [29]. Ates-Oz *et al*. (Protzer lab, Technical University of Munich) identified the co-stimulatory molecule 4-1BB as a potential therapeutic target, as it is expressed on T cells from mice with high-level HBV replication. Combination therapy of TherVacB with monoclonal antibodies activating 4-1BB improved CD8 T cell functionality and antiviral efficacy in high-titre HBV-carrier mice. These results provide new insights into T cell failure during chronic high-titre HBV infection but introduce a novel target for vaccine optimisation. Kosinska *et al*. (Protzer lab, Technical University of Munich/Helmholtz Munich) demonstrated that siRNA knockdown of HBV transcripts and the inhibitory ligand PD-L1 synergistically enhanced the efficacy of TherVacB in an AAV-HBV mouse model. Triple combination treatment significantly increased vaccine-elicited immunity and suppressed HBsAg and HBeAg.

Another approach to stimulate exhausted CD8^+^ T cells in chronic HBV infection was presented by Andreata *et al*. (Iannacone lab, San Raffaele Scientific Institute), who developed a *cis*-targeted fusion protein that activates the IL-2 pathway in CD8^+^ T cells (CD8-IL2). Importantly, CD8-IL2 significantly and specifically increased the number of HBV-reactive CD8^+^ T cells in the liver that showed increased expression of the effector molecules IFN-γ and granzyme B. CD8-IL2 therapy was associated with strong antiviral activity, with substantial reductions in serum HBV DNA, HBeAg and HBsAg, along with the frequency of HBc-expressing hepatocytes in the liver. The specific effect on CD8^+^ T cells was confirmed in cynomolgus monkeys, supporting the development of CD8-IL2 as a novel immunotherapeutic approach for the treatment of CHB.

## Antiviral therapies and preclinical models for drug evaluation

A breakthrough for the treatment of HDV was the approval of bulevirtide (BLV) (also known at Myrcludex B or hepcludex) [30] in some countries. BLV is derived from the myristoylated preS1 sequence (amino acids 1–48) and targets NTCP and blocks its interaction with HBV or HDV. To overcome the drawback of regular BLV injections, several groups have developed small molecules that block HBV entry. Windisch *et al*. (Assembly Bioscience, Inc) presented a preclinical evaluation of a class of molecules that inhibit NTCP-dependent HBV and HDV uptake. Meuleman *et al*. (Meuleman lab, Ghent University) characterised orally bioavailable NTCP antagonists that inhibit HBV infection of human liver chimaeric mice when administered prophylactically. Richardt *et al*. (Urban lab, University of Heidelberg) reported on the antiviral efficacy of bile acid–peptide conjugates and Funaki *et al*. (Yamashita lab, Kanazawa University Hospital) identified furoquinoline alkaloids as novel entry inhibitors against HBV and HDV infection. Building on the earlier observation that epidermal growth factor receptor (EGFR) can mediate NTCP-mediated viral internalisation, Kusunoki *et al*. (Watashi Lab, National Institute of Infectious Disease) identified compounds that block HBV infection by targeting the EGFR–NTCP interaction. Lempp *et al*. (VIR Biotechnology, USA) reported on the efficacy of VIR-2218, an RNAi therapeutic designed to knock down HBV transcripts and VIR-3434, an anti-HBsAg neutralizing monoclonal antibody blocking viral entry and reducing circulating HBsAg. Both compounds exhibited antiviral efficacy against HDV through multiple modes of action in cell culture and humanised mouse model and combination therapy is being tested in clinical trials.

Several CRISPR/Cas DNA editing-based therapeutics targeting cccDNA and/or viral integrants are under preclinical development. To improve the gene editing efficiency and accuracy of Cas9, Zhao *et al*. (Guo lab, Baruch S. Blumberg Institute and GeneLancet Life Sciences, Inc.) developed chemically ligated guide RNAs. The LgRNA/CRISPR-Cas9 disrupted the function of targeted viral genes with high precision and reduced HBV RNAs through unknown mechanisms. In an orthogonal approach McCoullough *et al*. (Revill/Littlejohn lab, VIDRL, RMH at the Peter Doherty Institute for Infection and Immunity) provided evidence that the RNA-guided RNA nuclease Cas13b in conjunction with small guide RNAs targeting conserved regions in the HBV genome suppresses HBsAg expression in cell culture model systems.

Anglero-Rodriguez and colleagues (Chroma Medicine and Tiget/INGM) showed that epigenetic regulation of the HBV genome led to sustained suppression of HBV antigens, with promising murine studies leading to elimination of HBsAg in 5/6 of mice tested. Although the method of epigenetic regulation was not defined, this study shows the potential for epigenetic targeting as a novel approach towards HBV functional cure.

HBV capsid assembly modulators (CAMs) can block multiple steps in the viral life cycle, including capsid trafficking to the nucleus, reverse transcription, pgRNA and polymerase protein co-packaging [31]. Strick-Marchand *et al*. (Institut Pasteur Paris) provided an update on GLP26 [32] treatment using an immunocompetent humanised mouse model, co-engrafted with human liver and a humanised immune system. Two months of GLP26 treatment reduced viraemia and viral antigens associated with enhanced immune responses and potentially improved outcomes off-therapy. In turn, Zlotnick *et al*. (Zlontnick lab, Indiana University) identified novel small-molecule antagonists of HBc that could disrupt HBV gene expression. In terms of selecting the optimal biomarkers for assessing the efficacy of new antiviral treatments, Pfefferkorn *et al*. (van Bommel lab, Leipzig University Medical Center) compared anti-HBc levels, HBsAg, HBV DNA, and HBV RNA to predict the treatment response to Peg-IFN. In this retrospective cohort study, individuals with a high anti-HBc titre (>4 log IU ml^−1^) at baseline were associated with a higher probability of achieving HBeAg seroclearance and virological response (HBV DNA <2000 IU ml^−1^) compared to those with anti-HBc levels <2 log IU ml^−1^ (59.1 and 72.7 % versus 0 and 28.6 %). Interestingly, the correlation between baseline anti-HBc levels and off-therapy virological response was reversed in HBeAg-negative CHB patients receiving Peg-IFN. The serum anti-HBc levels in HBeAg-positive CHB patients may serve as a marker for immune control, but further investigation is needed.

## HDV update

HBV was thought to be the only helper virus associated with HDV, however, recent studies identified divergent HDV-like viruses in fishes, birds, amphibians and invertebrates [33]. Fallon *et al*. (Weller lab, University of Utah) detected HDV RNA and HDAg in the salivary gland of patients with Sjogren’s syndrome, a chronic autoimmune disease. HBV markers (HBsAg or anti-HBs) were not detected in these patients, suggesting HBV-independent mechanisms of HDV infection [34]. Khalfi *et al*. (Majzoub lab, University of Montpellier) investigated the replication of HDV, rodent (RDeV) and snake deltavirus (SDeV) infectious clones in a variety of animal cell lines and in a mouse model, concluding that there was potential for cross-species transmission between members of the *Kolmioviridae*, raising questions about HDV origin and evolution. Tomba-Ngangas *et al*. (Le Gal lab, Hopital Avicenne) reported that the introduction of HDV into humans occurred around 5500 years BCE, suggesting a neolithic origin. HDV genotype 1, which represents the most ancient genotype, was dispersed in Central Africa to Eurasia, leading to worldwide dissemination.

HDV infection induces a robust IFN response that does not restrict RNA replication; however, exogeneous IFN inhibits HDV infection and the mechanisms underlying the differences in IFN response sensitivity are not known. Gnouamozi (Urban Lab, University of Heidelberg) presented comparative data on HDV-like agents in woodchuck (WoDV) and deer (DeDV) and showed that these agents do not express farnelysated viral antigens. Farnesylation of HDV large antigen (l-HDAg) is required for interaction with HBV surface proteins and HDV particle genesis. The authors presented data showing that WoDV and DeDV genomes could be packaged in cells expressing HBV surface antigens and l-HDAg. Importantly, WoDV and DeDV induced minor IFN responses in HepaRG-NTCP and Delta RNA replication was resistant to exogenous IFN treatment. l-HDAg is prenylated and Bauer *et al*. (Urban lab, University of Heidelberg) showed that blocking prenylation induced HDV-associated cell death through an MDA5-dependent pathway. Whether these post-translational modifications can be harnessed for therapeutic approaches requires study.

Chronic HDV is the most severe and rapidly progressive form of viral hepatitis. Allweiss *et al*. (Dandri lab, University Medical Center Hamburg-Eppendorf) studied a cohort of HBV/HDV-co-infected patients and analysed paired liver biopsies taken before and after 48 weeks of BLV treatment and examined the effect of BLV on HBV and HDV infection of immunodeficient humanised mice. BLV treatment reduced interferon-stimulated gene (ISG) and chemokine gene expression in co-infected patients and mice, suggesting that HDV is the primary factor triggering innate immune activation. Pfefferkorn *et al*. (van Bommel lab, Leipzig University Medical Center) showed that lower levels of MHBs may be a predictive biomarker for response to treatment with either BLV or with the immune modulator PEG-IFN-alpha, in patients with CHD. Chi *et al*. (Dao Thi lab, University Hospital Heidelberg) established a novel HDV infection model using human pluripotent stem cell-derived hepatocyte-like cells (HLCs). HLCs are permissive for different HDV genotypes and upon co-infection with HBV or ectopic HBsAg expression support the entire HDV life cycle. A small siRNA screen identified CD63 as a potential co-factor of HDV infection. Lin *et al*. (Chen lab, National Taiwan University) studied the impact of m^6^A modifications within the HDV genome on the replicative life cycle. METTL3-associated N6-methyladenine modification of HDV RNA at position A868 is required for the secretion of HDV particles but not for replication. Knock out of METTL3 or inhibition by a small-molecule inhibitor reduced HBV packaging. Fonte *et al*. (presented by Levrero, Levrero lab, Cancer Research Center of Lyon) investigated the antiviral activity of the nucleic acid polymer REP 2139 [35] in HDV-infected HepG2-NTCP cells and human hepatocytes. REP 2139 inhibited HDV replication by blocking the interaction of HDV RNA with HDAg, thereby preventing the formation of a ribonucleoprotein complex. These antiviral effects may explain the rapid decline of HDV RNA versus HBsAg observed in patients treated with REP 2139.

## Conclusions

Since the discovery of the Australia antigen in 1965 [36], subsequently determined to be the HBV envelope protein, considerable advances have been made in our understanding of HBV biology. Many of the discoveries were first presented and critiqued at the International HBV Meeting and some have been commercialised, as exemplified by Myrcludex B, which is licensed for the treatment of HDV infection. The 2023 Meeting was no exception and presented new findings on many aspects of HBV and HDV biology that we have highlighted in this article. However, gaps remain in our understanding of these small but impactful viruses (Table 1), particularly regarding the biogenesis and maintenance of the cccDNA reservoir, and manipulation of host immune responses, allowing viral persistence and development of end-stage liver disease. These gaps will be addressed at future International HBV Meetings, as we progress towards the curative therapies necessary to eliminate HBV- and HDV-related disease. To this end, the International HBV Meeting is committed to ensuring that the impacted community is represented and heard. To create the opportunity for learning and engagement between the scientific and patient communities, the Hepatitis B Foundation (HBF, USA) and the International Coalition to Eliminate Hepatitis B (ICE-HBV) co-host the Hepatitis B Community Forum each year. This forum focuses on progress towards HBV elimination and highlights the lived experiences, challenges, preferences, and needs of people living with chronic hepatitis B. In Kobe, the discussion included the challenges of medication access, fear and anxiety, stigma, discrimination, and highlighted the power of patient engagement.

The Meeting encourages and funds the participation of young and emerging researchers, who comprise many of the presenters. To this end, the 2023 Meeting commenced with the inaugural Emerging Researcher Workshop (organised by the HBF, USA). This interactive session was developed by the HBF Emerging Scientific and Medical Advisory Board to help early and mid-career researchers network with the goal of enhancing a sense of community and driving collaborations in the field. Small discussion groups were coordinated by junior researchers to brainstorm new research approaches in a casual, peer-led environment. These emerging investigators will be critical to addressing the considerable knowledge gaps and this forum will be an important feature of future annual Meetings. After all, it was Baruch Blumberg, who discovered HBV, who said that ‘Science is a forward looking endeavour’. Such forward thinking will be critical to the development of curative HBV and HDV therapies. The Meeting concluded with the ICE-HBV Symposium, an annual feature of the International HBV Meeting since 2019, with speakers from industry and academia focusing on the determinants of sustained HBV and HDV suppression in the setting of newly emerging RNA therapies.

## supplementary material

10.1099/jgv.0.001978 Uncited Supplementary Material 1.

## Abbreviations

cccDNA: covalently closed circular DNA
CHB: Chronic Hepatitis B
HBV: hepatitis B virus
HCC: hepatocellular carcinoma
NTCP: Sodium taurocholate co-transporting polypeptide
pgRNA: pregenomic RNA
RT: reverse transcriptase
WHV: woodchuck hepatits virus
